# Dietary Intake and Associated Factors in Long-Term Care Homes in Southeast Spain

**DOI:** 10.3390/nu11020266

**Published:** 2019-01-25

**Authors:** Ana Isabel Rodríguez-Rejón, María Dolores Ruiz-López, Reyes Artacho

**Affiliations:** 1 Department of Nutrition and Food Sciences, Faculty of Pharmacy, University of Granada, 18071 Granada, Spain; anarore@ugr.es (A.I.R.-R.); rartacho@ugr.es (R.A.); 2 Institute of Nutrition and Food Technology, University of Granada, 18100 Granada, Spain

**Keywords:** nursing homes, older adults, dietary intake, nutrients, protein, sarcopenia, nutrition assessment

## Abstract

Diet is a key modifiable factor in the management of malnutrition and age-related diseases such as sarcopenia, an important issue in long-term care homes. The objectives of this study were to evaluate the dietary intake of residents, define dietary patterns, and analyze their association with sex, diet texture, nutritional status, and the presence of sarcopenia. Intake was assessed by the precise weighing method, dietary patterns were defined a posteriori by cluster analysis, and nutritional status and sarcopenia were evaluated by applying the MNA-SF test and EWGSOP algorithm, respectively. A regular diet was consumed by 63% of participants; 56% were at risk of malnutrition and 63% were diagnosed with sarcopenia. Intake of potassium, magnesium, zinc, iodine, vitamin D, E, folic acid, and fiber was low in >80% of participants. Protein intake was <1 g/kg/day in 56% of participants and <25 g/meal in 100%. Two dietary patterns were identified, but neither fully met recommendations. The risk of a poorer diet was higher in females and residents with sarcopenia and was lower in those consuming regular diets. In conclusion, action is required to improve the inadequate nutritional intake of long-term care residents.

## 1. Introduction

The improvement of nutrition in long-term care (LTC) homes is an important research priority [1]. Diet is a key modifiable factor in the management of malnutrition [2,3] and age-related diseases, such as sarcopenia, an important issue in long-term homes [4]. Sarcopenia, the loss of muscle mass and muscle function associated with age [5], is associated with a low intake of energy and nutrients (e.g., protein, specific micronutrients), which appears to influence muscle mass (muscle quantity and quality) and function (strength and walking speed) [6,7,8,9,10]. Given that nutritional deficiencies do not generate clinical manifestations during early stages, monitoring of the dietary intake of residents is recommended to prevent severe deficiencies [11]. Various dietary assessment methods have been used to monitor dietary intake in institutionalized elderly people, but the precise weighing technique is established as the gold standard approach [12]. Few studies have undertaken the weighing of food because of the considerable effort and time required [13]; however, the relationship between dietary intake and health is complex, and accurate knowledge of the whole diet allows exploration of the health effects of interactions among food components [14]. Available statistical approaches to identify dietary patterns from intake data include principal component analysis, exploratory factor analysis, and cluster analysis. Cluster analysis defines patterns a posteriori, establishing subgroups of individuals with similar mean dietary intakes [15].

Malnutrition and sarcopenia are known to be common problems in LTC homes; however, there has been inadequate detailed research on the dietary intake of the residents, and the aim of the present study was to contribute further evidence in this regard. Therefore, the objectives of this study were: (1) to evaluate the dietary intake of institutionalized elderly people by the precise weighing method, (2) to characterize the study population according to dietary patterns defined by cluster analysis, and (3) to analyze the association of dietary patterns with sex, texture of diet, nutritional status, and the presence of sarcopenia.

## 2. Materials and Methods 

### 2.1. Study Design and Recruitment

This research was part of a cross-sectional study called the Granada Sarcopenia Study, which included a representative sample of permanent residents in three randomly selected LTC homes for older adults in Granada province (Southeast Spain). The recruitment and assessment procedures have been described elsewhere [16]. Inclusion criteria were: age ≥ 70 years, residence in the home for ≥3 months, stable medical condition, and written informed consent to participation from the resident or surrogate decision-maker. Exclusion criteria were: wearing a pacemaker, terminal state, receipt of palliative care, difficult or dangerous behavior, or the presence of medical or other problems preventing participation. Agreement to participation was obtained from directors of the LTC homes, and the study protocol was approved by the Ethics Committee of the University of Granada (Spain). 

### 2.2. Data Collection

Data were gathered by a qualified and previously trained dietician-nutritionist and level I anthropometrist (A.R-R.) certified by the International Society for the Advancement of Kinanthropometry (ISAK). Information was collected from LTC home records on sex, age, schooling, monthly income, admission date, and medical history. Participants were weighed using chair-scales (Seca 952; 0.1 kg), and their height was measured with their back against a wall or, when this was not possible, it was estimated from a standard formula based on knee height [17]. The body mass index (BMI) was calculated as weight/height*^2^*. A caliper (Innovare, Cescorf, Spain; 1 mm) was used to measure tricipital skinfold thickness (TST) and a flexible measuring tape (Cescorf, Spain; 1 mm) to measure mid-upper arm circumference (MUAC) and calf circumference (CC), calculating the mid-upper arm muscle circumference (MUAMC) as follows: *MUAMC = MUAC (in cm) − (0.314 x TST [in mm]*). The Barthel Index was used for the evaluation of daily living activities [18], the Lawton and Brody test for instrumental activities of daily living [19], the Functional Ambulation Classification (FAC) for the need for ambulation assistance [20], and the Pfeiffer test for cognitive status [21]. The mini-nutritional assessment short form test (MNA-SF) was used to assess nutritional status [22], considering a score of 12–14 points to indicate normal nutritional status, 8–11 points a risk of malnutrition, and <8 points malnutrition. Sarcopenia was diagnosed according to criteria of the European Working Group on Sarcopenia in Older People (EWGSOP) criteria [5], measuring muscle mass with an impedance meter, muscle strength using a Grip-D hand grip dynamometer, and gait speed (m/s) along a 4-meter course.

### 2.3. Dietary Assessment

Food intake data were collected for seven consecutive days (including a weekend) by the precise weighing method, weighing every portion served to individuals and the amount left on the plates at each meal [12]. The Nutrire® computer program was used for nutritional evaluation of the food consumed by each participant and for assessment of the nutritional composition of the dishes offered in different menus [23]. Results were compared with Dietary Reference Intakes (DRIs) for ≥70-year-olds [24,25], considering the Estimated Average Requirement (EAR) or, when EAR was not available, the Adequate Intake (AI). The Recommended Dietary Allowance (RDA) was also used for protein intake evaluation, alongside recommendations of the European Society for Clinical Nutrition and Metabolism (ESPEN) and PROT-AGE group [26,27]. The PROT-AGE group makes recommendations not only for the total daily protein intake but also for the minimum protein content of each main meal. The diet of each resident was classified according to its texture as regular (normal-texture), puréed, or mixed, assigning non-puréed foods with modified texture (soft or between soft and regular) to the “mixed” category.

### 2.4. Statistical Analysis

In a descriptive analysis of participants’ characteristics, categorical variables were expressed as frequencies and percentages and continuous variables as means with standard deviations. Results were stratified by sex. 

Mixed linear regression models were used to analyze nutrient data gathered for each meal consumed during 7 days, obtaining the marginal means adjusted for sex and the statistical significance of differences [28,29]. We also calculated the percentage of participants with intakes of micronutrients below the corresponding DRIs. 

The dietary intake of the population was characterized by cluster analysis, using two-stage clustering [30,31] to identify dietary patterns. Cluster selection was based on the Bayesian information criterion (BIC) and considering a silhouette coefficient > 0.4 in order to maximize the quality and the validity of the consistency of selected clusters [32].

Next, logistic multivariate analysis was conducted for the association of sex, texture of diet, nutritional status, and presence of sarcopenia with dietary patterns, verifying the goodness-of-fit of the model by using the BIC, residual analysis, and a receiver operating characteristics (ROC) curve for the estimated predictions [33,34].

SPSS version 25 was used for statistical analyses, considering a 5% significance level in all tests. 

## 3. Results

Table 1 displays the characteristics of the 249 participants (187 females and 62 males) who met eligibility criteria and were included in the study. The mean ± SD age was 84.9 ± 6.7 years, the socioeconomic level was predominantly low, and the majority suffered from moderate or severe functional and cognitive impairment. A regular (normal-texture) diet was consumed by 63% of participants; 56% of participants were at risk of malnutrition), and 63% were diagnosed with sarcopenia. 

Table 2 exhibits the mean dietary intakes of participants over a 7-day period by sex as measured by the precise weighing method and compares them with DRIs. In both sexes, mean intakes were below dietary recommendations for fiber, potassium, calcium, magnesium, zinc, iodine, vitamins D, E, B_3_, and B_6_, and folic acid. Intakes below dietary recommendations were also observed for selenium in females and for vitamin B_1_ in males. 

**Figure 1:**
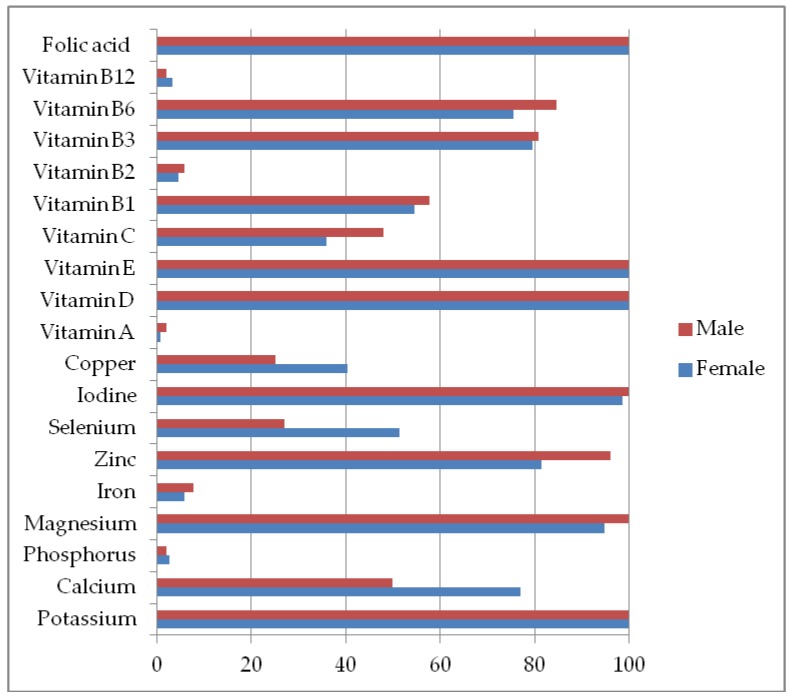
Percentage of participants not reaching the Dietary Reference Intakes for micronutrients.

As shown in Figure 1, the intake of potassium, magnesium, zinc, iodine, vitamin D, E, and folic acid was low in >80 % of the females and males, the intake of vitamins B_6_ and B_3_ was low in >80 % of the males and the intake of fiber was low in 89% of the females and 100% of the males.

**Figure 2:**
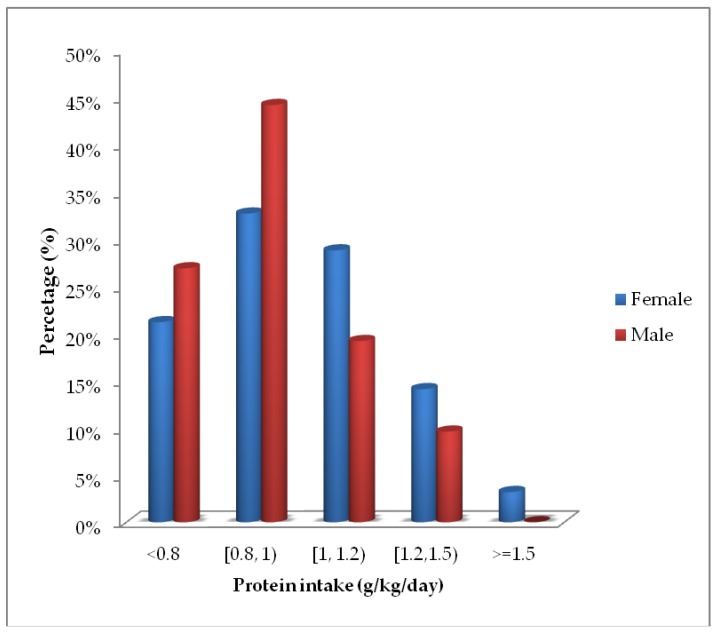
Percentage of female and male residents who did not reach the Recommended Dietary Allowance (RDA) for protein and the percentage that met ESPEN and PROT-AGE group recommendations in g/kg weight/day.

The mean daily intake of protein per kg of body weight was 1.00 (95% CI: 0.96, 1.03) g in females and 0.92 (95% CI: 0.85, 0.98) g in males. Figure 2 depicts the percentage of residents of each sex that did not reach the RDA for protein (0.8 g/kg/day) and the percentage that met recommendations of the ESPEN and PROT-AGE group (1–1.2 g/kg/day and 1.2–1.5 g/kg/day). Protein intake was <0.8 g/kg/day in 23% of participants, 0.8–1 g/kg/day by 36%, 1–1.2 g/kg/day in 26%, 1.2–1.5 g/kg/day in 13%, and >1.5 g/kg/day in 2%.

**Figure 3:**
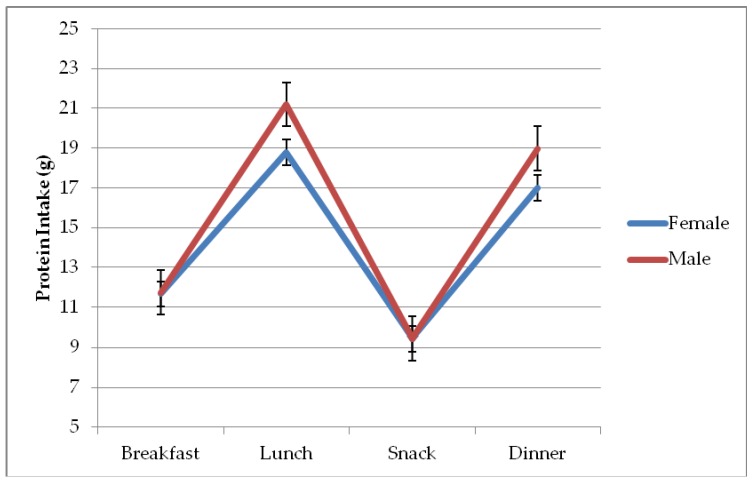
Protein intake of participants in each main meal by sex.

Figure 3 depicts the differences in mean protein intake among the different meals consumed in a day, being 11.66 (95% CI: 11.02, 12.29) g for females and 11.73 (95% CI: 10.63, 12.84) g for males at breakfast, 18.80 (95% CI: 18.16, 19.43) g for females and 21.20 (95% CI: 20.09, 22.30) g for males at lunch; 9.42 (95% CI: 8.78, 10.06) g for females and 9.44 (95% CI: 8.33,10.54) g for males at afternoon snack; and 17.00 (95% CI: 16.36, 17.64) g for females and 18.98 (95% CI: 17.87, 20.08) g for males at evening meal.

The cluster analysis derived in two dietary patterns, with one (designated "poorer diet") characterized by lower intakes in comparison to the other (designated "better diet"). Table 3 lists the intake variables in order of their importance for the identification of dietary patterns and gives the mean value for these variables in each of the two patterns identified. Although the nutritional quality was better in one dietary pattern than in the other, the mean intake of folic acid, potassium, energy, iron, lipids, monounsaturated fatty acids (MUFA), protein, MUFA C18:1, cholesterol, vitamin B_2_, phosphorous, zinc, vitamin B_12_, vitamin E, and magnesium was below recommendations in both of them.

As it is shown in Table 4, the results of the predictive logistic analysis revealed significant effects on dietary pattern (dependent variable: poorer vs. better diet) of sex, texture of diet, and sarcopenia (independent variables). The risk of a poorer diet was 3.2-fold higher for the females than for the males (*p* = 0.005), 4.6-fold lower for those consuming a regular versus mixed diet (*p* < 0.001) and 2.79-fold higher in participants with versus without sarcopenia (*p* = 0.004). There was no significant difference in risk between puréed and mixed diets or between regular and puréed diets. Likewise, no significant effect was found for nutritional status. 

The results of predictive classification as a function of the variables included in the model show that 74.5% of patients were correctly classified in the corresponding pattern. The area under the ROC curve was 0.79 (95 % CI: 0.73, 0.86), indicating the good fit of the predictive model. 

## 4. Discussion

In this study, the precise weighing method was used to analyze the diet of LTC residents who were generally characterized by a high age, low socioeconomic level, and frequent functional and cognitive impairment. The main finding was of an intake below dietary recommendations of protein, fiber, and certain vitamins (vitamins D, E, B_3_, and B_6_, and folic acid), and minerals (potassium, calcium, magnesium, zinc, iodine) in both sexes and of selenium in females and vitamin B1 in males.

Comparisons with findings of the few previous studies in LTC homes are hampered by differences in dietary assessment methods, dietary recommendations considered, and databases used [11,35,36,37,38]. However, although results vary among studies, the observation of inadequate intakes in institutionalized elderly people has been a frequent finding.

The protein intake of 22.6% of these residents was below the RDA (0.8 g/kg/day), and this in itself may be inadequate to maintain muscle health in the elderly [26,27,39,40]. The ESPEN and PROT-AGE group recommend 1–1.2 g/kg/day, an amount consumed by 26% of the study population, and a higher intake of 1.2–1.5 g/kg/day for those with acute or chronic disease, which was consumed by 13% of participants [26,27]. With regard to the distribution of this intake during the day, the PROT-AGE group proposed that elderly people should consume 25–30 g at each main meal (breakfast, lunch, evening) [26], and other authors recommended an intake of >30 g per main meal to maintain muscle mass while controlling fat mass [40]. These recommendations for the distribution of protein intake were not met in the present study. The largest protein intake was at lunch [18.80 [95% CI: 18.16, 19.43] g for females and 21.20 [95% CI: 20.09, 22.30] g for males). There has been limited research on this issue, with one study of older adults in a rehabilitation center finding that protein recommendations per meal were usually not met at breakfast or lunch except when certain foods were substituted by protein-enriched products [41]. 

The residents in this study were considered to have a “poorer” or “better” diet according to their dietary pattern as established by cluster analysis. The results of the logistic model show a significant association of sex, diet texture, and the presence of sarcopenia with dietary patterns. With respect to the texture of diet, participants with a regular diet had significantly lower risk of a poorer versus those with a mixed diet. Those who consumed a puréed diet had a higher risk of a poorer diet, although the difference was not significant. In a previous study, we found that the menus offered in these LTC homes [23] were below the EAR or AI for the minerals potassium, magnesium, zinc, iodine, calcium, and selenium and for vitamins D, E, C, B_3_, and folate in some or all cases. The lowest energy and macro/micro-nutrient values were observed in the puréed menus. Given these findings, the nutrient intake of the residents was expected to be inadequate. The lower energy and nutrient values in puréed than regular menus may explain the significant association observed between diet texture and dietary pattern. 

Iuliano et al. [42] raised concerns about the nutritional value of menus offered by LTC homes in Australia, observing that the intake of residents was below national dietary recommendations for calcium, zinc, magnesium, potassium, folate, and dietary fiber and included excessive sodium (3-fold higher than recommendations) and sugars. In addition, many of the residents did not meet recommendations for energy or protein intake. An insufficient intake of nutrients by the elderly is frequently attributed to their lack of appetite [43], but the aforementioned study found that the inadequacy of the meals provided by the home played a more important role. With regard to the quality of the menus offered by homes, puréed diets are of special interest and concern due to the work involved in their preparation and their low nutritional content, as found in the present study. Keller et al. [2] highlighted the need to improve the nutritional quality of puréed food, Dahl et al. [44] concluded that puréed food prepared in diets in Canadian LTC homes contained inadequate levels of protein, and Vucea et al. [45] reported a significant association between the consumption of a soft or puréed diet and a higher risk of malnutrition. Importantly, the energy or nutrient requirements of individuals needing a texture-modified diet do not differ from those of people with the same age and sex except in the presence of disease [46]; therefore, a puréed menu should meet the same general dietary recommendations. The texture of the diet (e.g., puréed) was prescribed for each resident according to their needs by the physicians at the residences, who are usually responsible for designing these menus in Spain. The staff responsible for serving the food (servers or kitchen assistants) would at times take into account the appetite, preferences or aversions of residents.

The risk of a poorer diet was 2.79-fold higher in the participants diagnosed with sarcopenia than in those who were not. Given that 63 % of the residents had sarcopenia, deficiencies in the diet of residents are a matter of major concern. An optimal intake of nutrient intakes is essential for the prevention and treatment of sarcopenia [9,10,47,48]. Besides the relationship of this condition with the inadequate consumption of protein, recent studies have described the need for the intake of vitamin D, an appropriate omega 6/omega 3 ratio [49,50], selenium, calcium, and magnesium [6]. There has been less research interest in the influence of overall diet quality on sarcopenia until recently [51]. Robinson et al. [52] underlined the importance of healthier dietary patterns in elderly people to ensure an adequate intake of proteins, vitamin D, antioxidant nutrients, and omega 3 (eicosapentaenoic and docosahexaenoic acids), given their potential role in sarcopenia prevention and treatment. 

In regard to nutritional status, it was previously observed that energy, protein, and micronutrient intakes are frequently low in malnourished residents or in those at risk of malnutrition [13,38]. In the present study, the intake of nutrients was not only inadequate in residents who were malnourished or at risk of malnutrition but also in those with normal nutritional status. Other authors have observed a poor energy and vitamin intake in a large proportion of elderly people classified as having normal nutritional status [53]. These results support the need to evaluate the actual dietary intake of residents as well as assessing their nutritional status with instruments such as the MNA-SF.

The strengths of this study include the use of validated methodologies by trained professionals. The precise weighing method is considered the gold standard, and data were gathered on 7 consecutive days for each participant. Cluster analysis permitted the identification of dietary patterns, enabling evaluation of the dietary intake as a whole rather than particular components.

The main limitation was the loss of some data due to the severe cognitive and functional impairment of some participants. Food in addition to the meals provided by the home was consumed by some residents, risking an underestimation of their intake; however, the extra intake was taken into account when detected by researchers or home staff.

## 5. Conclusions

The dietary intake of institutionalized elderly people in this study did not meet nutritional recommendations. The total amount of protein consumed per day and per meal did not meet the guidelines of the ESPEN and PROT-AGE group. Sex, texture of diet, and sarcopenia were associated with dietary intake as a function of the dietary pattern identified by cluster analysis. The risk of a poorer diet was higher in females and residents with sarcopenia and was lower in those consuming regular diets. However, this risk was not significantly affected by the nutritional status of participants, and nutritional recommendations were not fully met by any residents, whether they received a poorer or better diet. According to these findings, the nutritional intake of the residents of LTC homes, an especially vulnerable population group, remains inadequate. It is, therefore, necessary for action to be taken to ensure a sufficient food intake by each and every resident. This requires the special attention of properly trained health care professionals and the provision of meals that meet dietary recommendations, taking particular account of scientific evidence published on the supply of proteins. 

## Figures and Tables

**Table 1:** Characteristics of the long-term care home residents.

	Total (*n* = 249) *X* ± SD or *N* (%)	Female (*n* = 187) *X* ± SD or *N* (%)	Male (*n* = 62) *X* ± SD or *N* (%)
Age (years)	84.9 ± 6.7	85.4 ± 6.6	83.3 ± 7.0
Level of education			
Illiterate	38 (15)	32 (17)	6 (10)
Writing and reading ability	139 (56)	107 (57)	32 (52)
At least primary schooling	64 (26)	44 (24)	20 (32)
University degree	8 (3)	4 (2)	4 (6)
Level of income (€/month)			
<500	9 (4)	7 (4)	2 (3)
500–1000	184 (74)	151 (81)	33 (53)
1000–1500	46 (18)	26 (14)	20 (32)
>1500	10 (4)	3 (2)	7 (12)
Weight (kg)	62.2 ± 14.4	59.5 ± 14.1	70.2 ± 12.3
BMI (kg/m^2^)	26.3 ± 5.4	26.3 ± 5.8	26.4 ± 4.2
CC (cm)	32.1 ± 4.9	32.0 ± 5.3	32.7 ± 3.2
MUAMC (cm)	21.8 ± 2.9	21.3 ± 2.8	23.4 ± 2.5
Barthel test			
Independent	8 (3)	3 (2)	5 (8)
Mild	10 (4)	5 (3)	5 (8)
Moderate	65 (26)	44 (23)	21 (34)
Severe	78 (32)	63 (34)	15 (24)
Total	87 (35)	71 (38)	16 (26)
Lawton and Brody test			
Moderate	9 (4)	7 (4)	2 (3)
Severe	51 (21)	33 (18)	18 (29)
Total	187 (75)	145 (78)	42 (68)
FAC	1.2 ± 1.6	1.0 ± 1.4	1.8 ± 1.9
Pfeiffer test			
Intact	50 (22)	31 (18)	19 (37)
Mild	33 (14)	25 (14)	8 (15)
Moderate	50 (22)	36 (20)	14 (27)
Severe	95 (42)	84 (48)	11 (21)
Texture of diet			
Regular	128 (63)	91 (59)	37 (72)
Puréed	27 (13)	23 (15)	4 (8)
Mixed	50 (24)	40 (26)	10 (20)
MNA-SF			
Normal nutritional status	67 (27)	40 (21)	27 (44)
At risk of malnutrition	139 (56)	113 (61)	26 (42)
Malnourished	43 (17)	34 (18)	9 (14)
Sarcopenia			
Yes	143 (63)	119 (68)	24 (46)
No	84 (37)	56 (32)	28 (54)

Notes: Results are expressed as means (*X*) with standard deviation (SD) or number of participants (*N*) with percentage (%). There was one loss to follow-up for the main variable and for some secondary variables due to the characteristics of participants, but less than 10% of data were lost for any variable. Abbreviations: BMI, body mass index; CC, calf circumference; FAC, Functional Ambulation Classification; MNA-SF, Mini Nutritional Assessment-Short Form; MUAMC, mid-upper arm muscle circumference.

**Table 2:** Nutrient intake in care home participants.

		Female		Male		
Nutrients	EAR	*X* (SE)	95% IC (lower bound; upper bound)	*X* (SE)	95% IC (lower bound; upper bound)	*P*
Total energy (kcal/day)		1542.86 (19.06)	1505.28; 1580.43	1706.38 (33.01)	1641.29; 1771.46	<0.001
Protein (g/day)		57.55 (0.76)	56.04; 59.06	62.05 (1.32)	59.44; 64.67	0.004
Protein, g/kg of BW	0.66	1.00 (0.02)	0.96; 1.03	0.92 (0.03)	0.85; 0.98	0.035
Carbohydrates (g/day)	100	208.98 (2.75)	203.55; 214.41	229.71 (4.77)	220.31; 239.11	<0.001
Fiber (g/day)	21F/30 M ^ǂ^	14.60 (0.35)	13.91; 15.29	15.00 (0.60)	13.81; 16.19	0.566
Lipids (g/day)		52.98 (1.17)	50.68; 55.28	59.92 (2.02)	55.94; 63.91	0.003
SFA (g/day)		14.34 (0.43)	13.50; 15.18	15.86(0.74)	14.41; 17.31	0.075
MUFA (g/day)		17.56(0.58)	16.42; 18.70	20.61 (1.00)	18.64; 22.58	0.009
MUFA C16:1 (g/day)		1.19 (0.04)	1.11; 1.27	1.32 (0.07)	1.18; 1.46	0.122
MUFA C18:1(g/day)		15.16 (0.53)	14.12; 16.21	18.11 (0.92)	16.30; 19.92	0.006
PUFA (g/day)		5.89 (0.24)	5.41; 6.37	6.60 (0.42)	5.78; 7.44	0.142
PUFA C18:2 (g/day)		4.90 (0.23)	4.46; 5.35	5.62 (0.39)	4.85; 6.39	0.113
PUFA C18:3 (g/day)		0.25 (0.01)	0.23; 0.27	0.29 (0.02)	0.26; 0.33	0.048
Cholesterol (mg/day)		203.83 (5.23)	193.51; 214.14	232.83 (9.07)	214.96; 250.71	0.006
Potassium (mg/day)	4700 ^ǂ^	1805.22 (27.33)	1751.34; 1859.09	1953.31 (47.34)	1859.98 ; 2046.64	0.007
Calcium (mg/day)	1000	927.86 (9.98)	908.19; 947.52	997.26 (17.28)	963.20; 1031.33	0.001
Phosphorus (mg/day)	580	1013.41 (13.04)	987.70; 1039.12	1093.64 (22.59)	1049.11 ; 1138.18	0.002
Magnesium (mg/day)	265 F/350 M	196.41 (2.95)	190.60; 202.22	208.18 (5.10)	198.11; 218.24	0.047
Iron (mg/day)	5 F/6 M	7.27 (0.13)	7.02; 7.52	8.00 (0.22)	7.56; 8.44	0.005
Zinc (mg/day)	6.8 F/9.4 M	5.64 (0.13)	5.38; 5.91	6.35 (0.23)	5.90; 6.81	0.009
Selenium (µg/day)	45	44.27 (1.48)	41.36; 47.18	51.78 (2.56)	46.74; 56.82	0.012
Iodine (µg/day)	95	29.89 (2.10)	25.75; 34.03	32.66 (3.64)	25.49; 39.83	0.511
Copper (µg/day)	700	775.43 (17.18)	741.56; 809.31	801.35 (29.77)	742.66; 860.05	0.452
Vitamin A (µg/day)	500 F/625 M	1306.26 (30.64)	1245.84; 1366.68	1413.06 (53.12)	1308.33 ; 1517.79	0.083
Vitamin D (µg/day)	10	1.37 (0.06)	1.25; 1.50	1.57 (0.11)	1.35; 1.78	0.126
Vitamin E (mg/day)	12	4.60 (0.17)	4.26; 4.94	5.61 (0.30)	5.02; 6.20	0.004
Vitamin C (mg/day)	60 F/75 M	71.85 (2.28)	67.36; 76.35	80.80 (3.95)	73.02; 88.59	0.051
Vitamin B_1_ (mg/day)	0.9 F/1 M	0.93 (0.02)	0.89; 0.96	0.98 (0.03)	0.91; 1.05	0.172
Vitamin B_2_ (mg/day)	0.9 F/1.1 M	1.30 (0.02)	1.27; 1.33	1.38 (0.03)	1.32; 1.44	0.017
Vitamin B_3_ (mg/day)	11 F/12 M	9.14 (0.21)	8.72; 9.56	9.66 (0.37)	8.94; 10.40	0.217
Vitamin B_6_ (mg/day)	1.3 F/1.4 M	1.16 (0.02)	1.11; 1.20	1.20 (0.04)	1.13; 1.27	0.316
Vitamin B_12_ (µg/day)	2	3.09 (0.06)	2.98; 3.20	3.48 (0.10)	3.28; 3.67	0.001
Folic acid (µg/day)	320	128.53 (2.54)	123.53; 133.53	142.79 (4.39)	134.12; 151.45	0.005

Note: Results are expressed as means (*X*) with standard error (SE) and 95% confidence interval (CI). Marginal means resulting from the mixed linear regression models are shown. Abbreviations: AI, adequate intake; BW, body weight; EAR, estimated average requirement; F, female; M, male; MUFA, monounsaturated fatty acids; PUFA, polyunsaturated fatty acids; SFA, saturated fatty acids. ^ǂ^ AI instead of EAR.

**Table 3:** Characterization of dietary patterns as a function of intake variables in order of importance.

		Poorer diet (*n* = 112)		Better Diet (*n* = 96)	
		*X* (SD)	95% CI	*X* (SD)	95% CI
Folic acid	1	110.66 (20.74)	(106.78–114.54)	157.01 (24.31)	(152.08–161.93)
Potassium	1	1611.79 (258.82)	(1563.33–1660.25)	2110.48 (221.04)	(2065.7–2155.27)
Energy	0.99	1419.63 (172)	(1387.42–1451.83)	1774.76 (174.96)	(1739.31–1810.21)
Iron	0.97	6.37 (1.22)	(6.14–6.6)	8.71 (1.08)	(8.49–8.93)
Lipid	0.97	44.95 (10.82)	(42.93–46.98)	66.07 (10.06)	(64.03–68.11)
Vitamin B_1_	0.91	0.78 (0.16)	(0.75–0.81)	1.12 (0.19)	(1.08–1.16)
MUFA	0.87	13.69 (5.33)	(12.69–14.68)	23.72 (5.38)	(22.63–24.81)
Protein	0.83	52.6 (7.44)	(51.2–53.99)	65.73 (7)	(64.31–67.14)
MUFA C18:1	0.83	11.73 (5)	(10.79–12.66)	20.76 (5.01)	(19.74–21.77)
Cholesterol	0.79	170.32 (53.34)	(160.33–180.3)	258.07 (46.06)	(248.73–267.4)
Vitamin B_2_	0.74	1.2 (0.16)	(1.17–1.23)	1.46 (0.16)	(1.43–1.5)
Phosphorous	0.73	934.98 (133.16)	(910.05–959.92)	1147.99 (122.66)	(1123.13–1172.84)
Zinc	0.7	4.84 (1.14)	(4.62–5.05)	6.97 (1.52)	(6.66–7.28)
Vitamin B_12_	0.68	2.76 (0.59)	(2.65–2.87)	3.67 (0.56)	(3.56–3.78)
Vitamin E	0.62	3.64 (1.62)	(3.33–3.94)	6.27 (1.91)	(5.88–6.66)
Magnesium	0.62	178.9 (29.35)	(173.4–184.39)	223.19 (30.47)	(217.01–229.36)
PUFA	0.57	4.44 (1.99)	(4.07–4.82)	7.96 (2.97)	(7.35–8.56)
Selenium	0.55	36.32 (15.14)	(33.49–39.16)	57.6 (15.81)	(54.39–60.8)
MUFA C16:1	0.53	0.96 (0.42)	(0.88–1.03)	1.53 (0.44)	(1.44–1.62)
PUFA C18:2	0.52	3.63 (1.92)	(3.27–3.98)	6.77 (2.77)	(6.21–7.33)
Vitamin C	0.5	59.55 (22.47)	(55.34–63.76)	90.98 (25.83)	(85.75–96.21)
Calcium	0.46	882.98 (108.08)	(862.74–903.22)	1017.72 (109.92)	(995.45–1039.99)
Vitamin B_6_	0.44	1.04 (0.24)	(0.99–1.08)	1.32 (0.22)	(1.27–1.36)
PUFA C18:3	0.43	0.2 (0.1)	(0.18–0.22)	0.33 (0.12)	(0.31–0.35)
Vitamin A	0.41	1152.95 (319.78)	(1093.08–1212.83)	1539.86 (349.54)	(1469.03–1610.68)
Vitamin B_3_	0.4	8.04 (2.24)	(7.62–8.46)	10.69 (2.45)	(10.2–11.19)
Fiber	0.38	12.75 (3.38)	(12.12–13.38)	16.97 (4.27)	(16.1–17.84)
SFA	0.36	12.41 (4.06)	(11.65–13.17)	17.4 (5.4)	(16.31–18.49)
Copper	0.35	689.34 (216.88)	(648.73–729.95)	888.03 (155.15)	(856.6–919.47)
Carbohydrates	0.25	201.17 (29.37)	(195.67–206.67)	229.31 (36.01)	(222.01–236.6)
Vitamin D	0.24	1.14 (0.67)	(1.01–1.26)	1.75 (0.81)	(1.58–1.91)
Iodine	0.17	22.8 (19.87)	(19.08–26.52)	39.64 (29.63)	(33.64–45.65)

Note: Results are expressed as means (*X*) with standard deviation (SD) and 95% confidence interval (CI). Abbreviations: MUFA, monounsaturated fatty acids; PUFA, polyunsaturated fatty acids; SFA, saturated fatty acids.

**Table 4:** Parameter estimates from the multivariate logistic regression model fitted to the dietary patterns.

	Parameter Estimates						
Poorer diet vs. Better diet		95% Wald Confidence interval for Exp(B)				Hypothesis Test	
Parameter	B	Exp(B)	Lower	Upper	Wald Chi-Square	Df	Sig.
Female vs. Male	1.165	3.205	1.416	7.253	7.808	1	0.005
Regular diet vs. Mix diet	−1.541	0.214	0.090	0.508	12.245	1	0.000
Puréed diet vs. Mix diet	−1.079	0.340	0.100	1.153	3.000	1	0.083
MNA-SF Normal vs. Malnourished	−1.045	0.352	0.111	1.113	3.161	1	0.075
MNA-SF At risk vs. Malnourished	−0.276	0.759	0.271	2.123	0.276	1	0.599
Sarcopenia vs. No sarcopenia	1.026	2.790	1.394	5.584	8.401	1	0.004

Note: Age was not a significant factor and did not improve the goodness of fit of the model (*p* > 0.1). MNA-SF: Mini Nutritional Assessment-Short Form.
